# Information recovery from low coverage whole-genome bisulfite sequencing

**DOI:** 10.1038/ncomms11306

**Published:** 2016-06-27

**Authors:** Emanuele Libertini, Simon C. Heath, Rifat A. Hamoudi, Marta Gut, Michael J. Ziller, Agata Czyz, Victor Ruotti, Hendrik G. Stunnenberg, Mattia Frontini, Willem H. Ouwehand, Alexander Meissner, Ivo G. Gut, Stephan Beck

**Affiliations:** 1 Medical Genomics, UCL Cancer Institute, University College London, London WC1E 6BT, UK; 2 Centro Nacional de Análisis Genómico (CNAG), Parc Científic de Barcelona, Torre I, 08028 Barcelona, Spain; 3 Division of Surgery and Interventional Science, University College London, London W1W 7EJ, UK; 4 Broad Institute of MIT and Harvard, Cambridge, Massachusetts 02142, USA; 5 Harvard Stem Cell Institute, Cambridge, Massachusetts 02138, USA; 6 Department of Stem Cell and Regenerative Biology, Harvard University, Cambridge, Massachusetts 02138, USA; 7 Illumina Inc., San Diego, California 92121, USA; 8 Department of Molecular Biology, Radboud University Nijmegen, Nijmegen 6525 GA, Netherlands; 9 Department of Haematology, University of Cambridge, Cambridge, CB2 0XY, UK; 10 National Health Service Blood and Transplant, Cambridge Biomedical Campus, Cambridge, CB2 0XY, UK; 11 British Heart Foundation Centre of Excellence, University of Cambridge, Cambridge, CB2 0QQ, UK; 12 Wellcome Trust Sanger Institute, Wellcome Trust Genome Campus, Hinxton, Cambridge, CB10 1SA, UK

## Abstract

The cost of whole-genome bisulfite sequencing (WGBS) remains a bottleneck for many studies and it is therefore imperative to extract as much information as possible from a given dataset. This is particularly important because even at the recommend 30X coverage for reference methylomes, up to 50% of high-resolution features such as differentially methylated positions (DMPs) cannot be called with current methods as determined by saturation analysis. To address this limitation, we have developed a tool that dynamically segments WGBS methylomes into blocks of comethylation (COMETs) from which lost information can be recovered in the form of differentially methylated COMETs (DMCs). Using this tool, we demonstrate recovery of ∼30% of the lost DMP information content as DMCs even at very low (5X) coverage. This constitutes twice the amount that can be recovered using an existing method based on differentially methylated regions (DMRs). In addition, we explored the relationship between COMETs and haplotypes in lymphoblastoid cell lines of African and European origin. Using best fit analysis, we show COMETs to be correlated in a population-specific manner, suggesting that this type of dynamic segmentation may be useful for integrated (epi)genome-wide association studies in the future.

## Abstract (web summary)

Here, Libertini and colleagues devise a computation tool that can analyze whole-genome bisulfite sequencing (WGBS) data to recover of ∼30% of the lost differential methylation position information. They use COMETgazer and COMETvintage to analyze 13 diffferent methylome data to demonstrate their performance.

## Graphical abstract

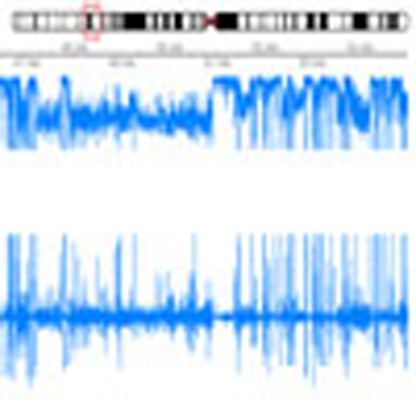

Whole-genome bisulfite sequencing (WGBS) is the method of choice for the generation of reference methylomes^1^,^2^,^3^ and increasingly being used in basic and clinical research as well^4^. To facilitate the complex analysis of such WGBS methylomes, a wide range of pipelines and algorithms has been developed with respect to cost, scale, resolution and biological questions^5^,^6^,^7^. Informed decisions on resource allocation need to be made to tailor the data analysis to the experimental design while taking into account the advantages and disadvantages of single CpG resolution profiling with WGBS, where methylation estimates are derived from a cell population or a single cell assay. In a separate study^8^, we conducted an assessment of sequencing coverage required for quantitative detection of resolution-dependent methylome features such as differentially methylated positions (DMPs e.g., dynamic CpG sites^9^), differentially methylated regions (DMRs, e.g., tissue or disease specific DMRs^10^) and blocks of comethylation (COMETs), described here. As part of this study, we found that the majority of publicly available methylomes are single replicate, restricting the statistical analysis to e.g., Fisher's Exact test without the ability of taking biological variation into account. More recent tools for the analysis of differences at single CpG sites use counts of methylated and unmethylated reads at any given site. MethylKit^11^, for example, uses the binomial distribution within a logistic regression framework, while several packages use beta-binomial assumptions for WGBS experiments including DSS^12^, MOABS^13^ and RADMeth^14^. BSmooth^10^ employs local-likelihood estimation for statistical smoothing in order to strengthen methylation inference at the regional (DMR) level—a technique which works particularly well if methylation differences are large (e.g., in cancer) or where multiple replicates were chosen over deep sequencing of single replicates.

Another area of recent advancement includes analyses based on patterns of comethylation which were first observed over short (∼1 kb) distances by chromosome-wide profiling^15^ and subsequently confirmed by WGBS^16^,^17^. More recently, similar types of analyses have been developed for the identification of regulatory regions using *methylSeekR*^18^, genetically controlled methylation clusters (GeMes^19^) and the visualisation of regional epigenome-wide association scan (EWAS) results^20^. Building on these advancements, we have developed COMETgazer, an algorithm for determining the stochastic oscillations of DNA methylation to dynamically segment entire methylomes into COMETs and COMETvintage to call DMCs. We then used these algorithms to assess their suitability to recover the ∼50% loss of DMP information observed in our methylome saturation analysis^8^ and to determine the relationship between COMET and haplotype block seizes.

## Results

### Oscillatory analysis for methylome segmentation

**Figure 1:**
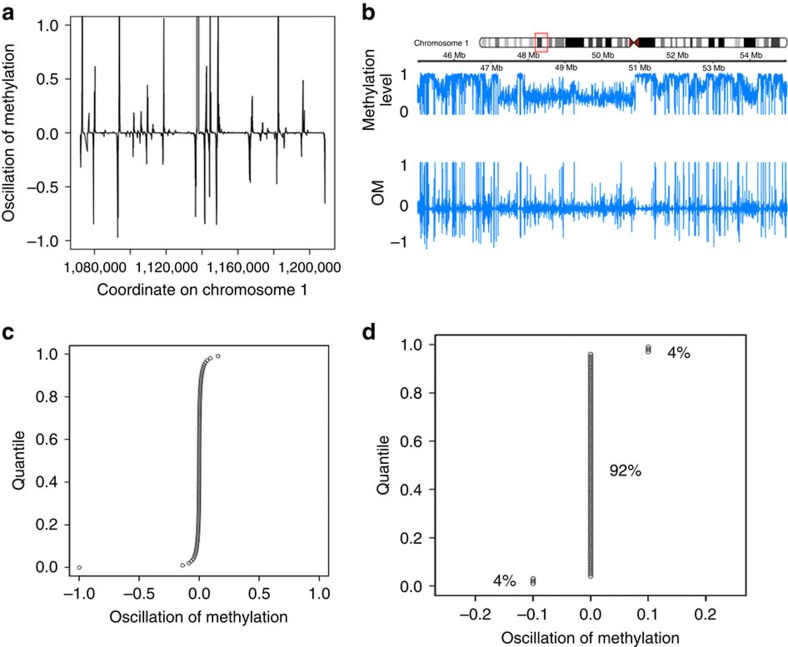
Relationship between methylation values and oscillation of methylation (OM) for chromosome 1 of M1. (**a**) Patterns of oscillations as estimated by OM. Values were scaled to 0–1. (**b**) Relationship between methylation value and OM distribution in a representative region of M1. Delta (OM) values were scaled to 0–1. (**c**) Quantile distribution of OM values. Most oscillations are around 0, significant oscillations represent a deviation from the co-methylation and are used to call the successive COMET boundaries. (**d**) Rounded quantile distribution of OM values. COMETs are called using the dynamic OMg threshold which is defined by significant deviations in the OM distribution, representing roughly 8% of the OM values for the methylomes included here.

**Figure 2:**
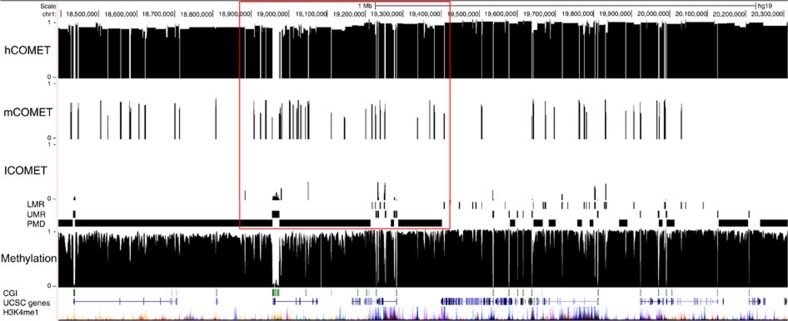
COMETgazer and MethylSeekR segmentation for M1 with corresponding methylation values. A red box highlights the fine-grained nature of COMET analysis in segmentation compared with features defined by MethylSeekR. This example region shows the COMET break-down of multiple MethylSeekR features.

First, we assessed the performance of COMETgazer and COMETvintage on 13 WGBS methylomes which are summarized in Supplementary Table 1 and Methods. All WGBS methylomes were segmented into consecutive COMETs following defined patterns of oscillation in methylation values (Supplementary Table 2). COMETs were calculated using varying oscillator of methylation grade (OMg) scores based on consecutive CpG methylation smoothed estimates. Analogous to the r^2^ measure^21^ which is commonly used to define linkage disequilibrium (LD) and haplotypes, OMg scores are used here to dynamically define COMETs. Figure 1 shows key features of COMET analysis. Oscillations in DNA methylation (OM) are defined as a continuous CpG density-independent K-period percentage difference series (Fig. 1a) based on the continuous smoothed methylation level estimate (Fig. 1b). The quantile distribution of OM values is analysed independently for each chromosome (e.g., chromosome 1 is shown in Fig. 1c). Most of the oscillations are around zero, and these define regions of co-methylation. Fragmentation in the methylome structure is defined as significant deviations in the quantile distribution used to call individual COMETs (Fig. 1d). COMETgazer segments the entire methylome into consecutive COMETs based on DNA methylation oscillations, which define regions of transition at fine-grained level. Given the need to quantitatively define fragmentation for differential co-methylation analysis (e.g., DMC analysis), methylomes need to be analysed at the highest possible resolution. To illustrate this point, we compared COMET analysis with *MethylSeekR* which was developed to identify active regulatory regions by segmenting methylomes into umethylated regions (UMRs), low-methylated regions (LMRs) and partially methylated domains (PMDs). Figure 2, for example, shows that PMDs have variable COMET content by fragmenting into multiple lowly (l) and medium (m) methylated COMETs and can even include highly (h) methylated ones. Together, the l- m- and hCOMETs are then used for sensitive identification of DMCs. Supplementary Table 3 summarizes the correlations between these features. PMDs have a correlation of 0.6 with hCOMETs but also include mCOMETs and lCOMETs. The latter have a relationship with UMRs and CGI (0.7 and 0.4 correlation, respectively). The segmentation obtained with COMETgazer is therefore substantially different to that from *MethylSeekR*. Compared to fixed regional thresholds used by *MethylSeekR*, COMETgazer uses dynamic segmentation parameters for CpG-wise processing of methylation values along chromosomes, allowing for higher resolution analysis of comethylation. Comparative analysis of M1, for example, reveals an average block size of ∼1,000 bp for COMETgazer compared to ∼25,000 bp for PMDs defined by *MethylSeekR* as regions of extended variable methylation, irrespective of methylation level. Figure 2 and Supplementary Fig. 2 show examples where PMDs span across CGIs and genes whereas the higher resolution COMETs may help to analyse the structure of these regions in more detail.

### Information recovery of methylome features

Next, we compared COMET/DMC and DMR analyses to assess the possible recovery of DMP information lost in dependence of coverage after downsampling IHEC replicate methylomes M7–10 against deep replicates M1–2 as part of a separate saturation analysis^8^. For each methylome, the number of iCGs, DMPs, DMRs, COMETs and DMCs was determined. Two established algorithms (BSmooth^10^ and RADmeth^14^) were used for DMR and DMP calling respectively, while COMETvintage was used for DMC calling (Supplementary Fig. 1, Methods). COMETvintage uses the COMET distributions (Supplementary Fig. 2) as a count matrix with fixed windows applying a negative binomial model to obtain DMCs.

**Figure 3:**
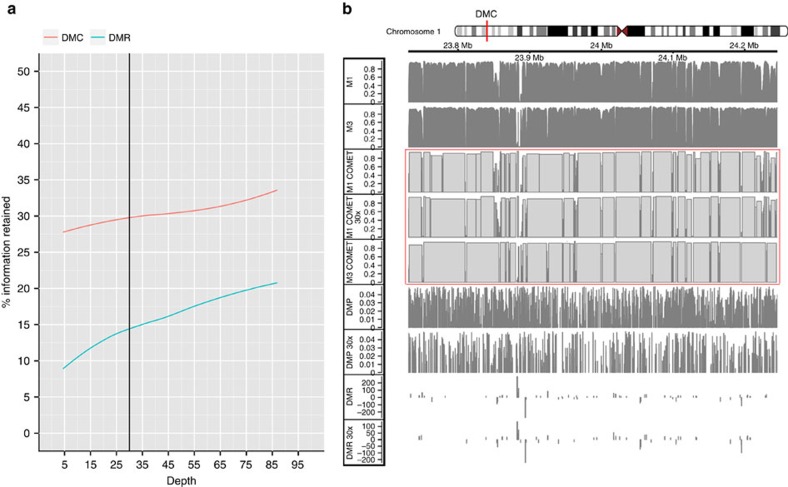
Information recovery by DMC analysis. (**a**) Semi-quantitative DMP content recovery rates for DMR and DMC analysis based on the results from the RADmeth replicate analysis. DMP calls were set at *P*<0.05 after BH adjustment. DMRs are typically short compared to DMCs which accounts for the difference in DMP counts. (**b**) Example DMC (boxed red) showing methylation level (tracks 1–2), COMETs (tracks 3–5), DMPs (tracks 6–7), and DMRs (tracks 8–9) for M1 at maximum and 30X coverage, as well as M3. DMP calls are shown as adjusted p values for differential methylation between M1–2 and M7–10. DMR values representing differential methylation between M1–2 and M7–10 correspond to a*reaStat*, a parameter of compound t-statistics for the included DMPs. COMET values correspond to the average value inside each block.

As our separate saturation analysis revealed that DMP calling at ∼30X coverage only captures ∼50% of DMPs in a replicate analysis^8^, we assessed whether part of the lost information could be recovered through the analysis of higher complexity features such as DMRs and DMCs (semi-quantitative DMP content recovery). For this, we developed a suite of novel algorithms (COMETgazer and COMETvintage) which are freely available at https://github.com/rifathamoudi/COMETgazer. Semi-quantitative DMP content recovery was measured by overlaps of significant features through smoothing as implemented in BSmooth^10^ for DMR analysis and measuring the breakage of COMETs by DMPs as implemented in COMETvintage for DMC analysis (Supplementary Fig. 1, Methods). The recovery using DMC analysis was on average 2.5-fold higher than for DMR analysis (Fig. 3a). DMC analysis recovered ∼35% of the estimated RADmeth DMPs at maximum coverage, and ∼30% at only 5X. In contrast, DMR analysis recovered only ∼20% of the DMPs at maximum coverage and ∼10% at 5X. The difference between DMR and DMC performance is most likely caused by individual DMPs disregarded by DMR callers but able to break COMETs and thus detected by COMETvintage. Figure 3b shows an example of a DMC between M1–2 and M7–10 created by fragmentation of COMETs. For comparison, the underlying DMPs and DMRs are also shown at maximum and 30X coverage. Calling of DMRs and COMETs at different coverage is highly reproducible (Supplementary Fig. 3).

### Relationship between co-methylation and haplotypes

**Figure 4:**
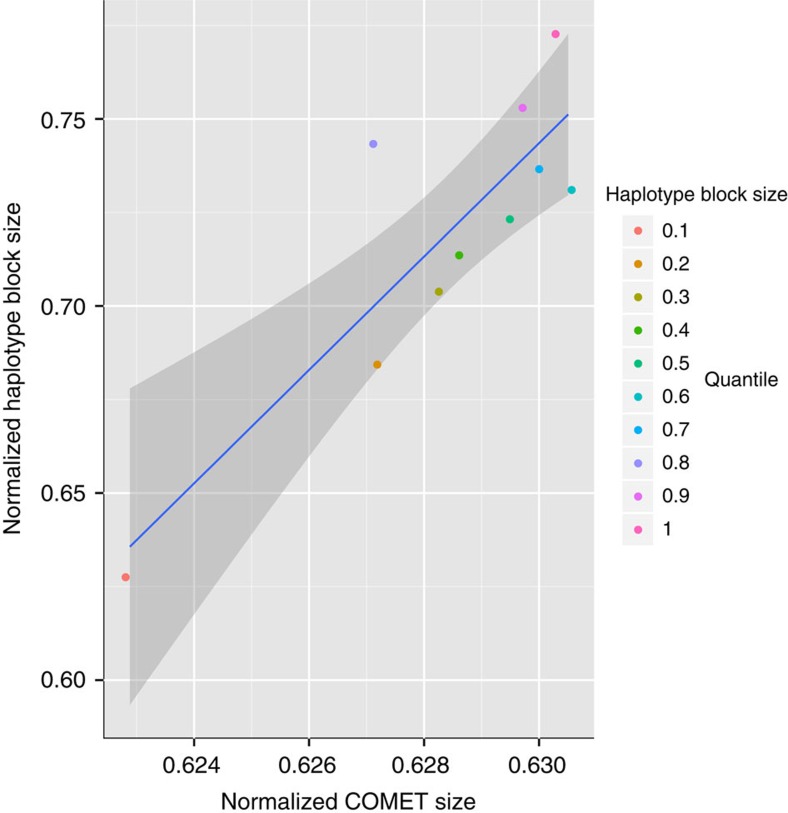
Correlation between African (YRU) haplotype blocks and YRU COMETs derived from M5. Median haplotype block size defined by r^2^>0.9 versus median COMET size defined by OMg=0.1. Data was tiled over fixed windows of 100,000 bp and scaled over 0–1 (Methods).

Finally, we explored the relationship between COMETs and haplotypes. As WGBS methylome data become available on a population-wide level, high complexity feature analysis such as COMETs may also offer a way to generate an epigenetic equivalent of the haplotype map (HapMap)^21^. To illustrate this potential, we generated a 37X methylome (M5) of an African (YRU) HapMap cell line (GM18507) with known linkage disequilibrium (LD) structure and compared the YRU-derived COMETs with the corresponding YRU haplotype blocks defined by LD (Figure 4, Supplementary Figure 5). A best fit analysis revealed high correlation (r=0.86, *P*-value=0.00112) for r^2^=0.9 and OMg=0.1 (Supplementary Table 4) which decreased as expected by 0.4% in significance when replacing YRU by a more distant and less fragmented European (CEU) haplotype (Supplementary Fig. 6). Taken together, these findings suggest a possible functional relationship between genetic and epigenetic (DNA methylation) variants in line with recent observations using related analyses^22^,^23^.

## Discussion

A recent saturation analysis of WGBS data revealed a major limitation for calling DMPs in methylomes generated at the recommended reference coverage of 30X (ref. 8). Using a novel approach of segmenting WGBS methylomes into COMETs for subsequent calling of DMCs, we present a solution that is able to recover approximately 30% of the lost DMP information content in the form of DMCs, doubling the recovery achievable to date by DMR analysis. However, our COMET/DMC analysis is not without limitation either. As for DMR analysis, DMP recovery by DMC analysis is not possible at single CpG level. For that, the corresponding DMRs and DMCs need to be subjected to additional targeted BS-seq for which a variety of methods are readily available^5^,^6^,^7^. However, as biological processes predominantly involve multiple and frequently clustered changes in CpG methylation^24^. DMR/DMC resolution will be adequate for many functional studies. An alternative solution would be to recover lost DMPs by imputation which proved highly successful for the recovery of single nucleotide polymorphisms (SNPs) in low-coverage whole-genome sequencing^25^. Towards this goal, a first method (ChromImpute) was recently developed and shown to be capable of imputing epigenomic maps with as little as 26% of supporting experimental data^26^. While the imputed data were similar to the observed experimental data and even surpassed them in consistency, multiple complementary data were required to impute any particular mark, e.g., it is currently not possible to impute DMPs from WGBS data alone.

In addition to DMP recovery, we show COMET analysis to complement low-resolution functional methylome studies using PMD analysis. The COMETgazer algorithm provides a fine-grained segmentation of the methylome which breaks down variable regions (and detects regions of transitions) with an average block size of ∼1,000 bp for COMETs compared to ∼25,000 bp for PMDs, facilitating the identification of novel regulatory elements such as promoters and enhancers within PMDs through differential comethylation using a negative binomial model. We propose that the oscillatory patterns of DNA methylation and the number of COMETs (the fragmentation) may be used as an additional metric to characterize epigenomes and are currently pursuing an integrative analysis with other epigenomic datasets including additional modalities (histone medications, RNAseq and HiSeq) from the International Human Epigenome Consortium (IHEC). A more speculative application of COMET analysis may be to harness it in the future for epigenome-wide association studies^27^ and the generation of an epigenetic equivalent of the haplotype map^28^. Although only based on two cell lines from African and European descent, our finding that the relationship between COMETs and haplotypes appears to be population-specific is certainly interesting and warrants further investigation once WGBS data become available on a population level.

## Methods

The key metrics of the methylomes used here are summarized in Supplementary Table 1 and further details are described below.

### Datasets

The M1 and M2 datasets have been deposited together into EGA under accession number EGAD00001001261. The M3 dataset was downloaded from GEO using accession numbers: GSM1112840 (M7), GSM1112841 (M8) GSM916051 (M9), GSM1112848 (M10), belonging to superseries GSE46644. The M4 dataset was downloaded from GEO series GSE17917 described in Lister *et al*.^29^ The M5 dataset has been deposited into GEO under accession number GSE66285. M6 was obtained from EGAD0000100673. M11 and M12 were obtained from GSM1112838 and GSM112842, respectively. M13 was downloaded from GSE17972.

### Data processing and analysis

*Read mapping*. Two reference sequences were prepared based on the hg19 human reference; reference_C2T had the C residues replaced by Ts, and reference_G2A had the Gs replaced by As. The sample preparation protocol followed ensures that reads from end 1 are from either of the original DNA strands, and are therefore generally C deficient (as unmethylated C residues are converted to T), and reads from end 2 are from the complement to the original strands and are therefore generally G deficient. The read data were fully converted prior to alignment, converting the remaining C's to T's in end 1, and converting G's to A's in end 2. The WGBS data was aligned using the GEM aligner (Kulis *et al*.)^30^ and (Marco-Sola *et al*.)^31^ allowing up to 4 mismatches from the reference. Uniquely mapping reads were selected where both read end1 mapped consistently, and no other consistent set of mappings for a read pair was found with the same number of mismatches. Duplicate read pairs were identified as read pairs mapping to the same position at both ends, and such pairs were merged to produce a consensus sequence for downstream analysis. Overlapping read pairs were handled by generating a single long read with the overlapping portion representing the consensus between the two ends. After read mapping, the reference sequence (C2T or G2A) that the read pair mapped to was recorded, and the original read data restored. Prior to further analysis, the 5 base pairs at the start of both read ends were trimmed since methylation estimation from these positions are unreliable due to the end repair procedure during sample preparation.

### Inference of genotype and methylation status

Genotype and methylation status were estimated simultaneously using software developed at the Centre Nacional d'Anàlisi Genòmica. A Bayesian model, taking into account the probability of under and over conversion and sequencing error, was used to estimate the joint posterior probability of genotype and methylation at each genomic site covered by at least 2 reads. The marginal posterior genotype probability was estimated by numerical integration of the joint posterior (using Gaussian quadrature). For sites where a single genotype presented >99% of the posterior distribution, the maximum likelihood estimate of the methylation and the standard error of the estimate conditional the most probable genotype were calculated. CpG calls for downstream analysis were produced from pairs of sites called as homozygous C followed by homozygous G with high confidence (posterior probability >0.99).

### Feature and saturation analysis

The feature and saturation analysis was conducted at University College London. All methylomes were analysed on autosomes only. Features of increasing complexity were defined and computed for the subsequent saturation analysis as follows: Informative CpG sites (iCGs) were defined as canonical CpGs of at least 8X read coverage. Coverage was calculated on median counts across all iCGs and shown as iCG saturation curve. Counts were independently and randomly downsampled for every CpG. Differentially methylated positions (DMPs) in replicate analysis were defined as iCGs of genome-wide significance (*P*<0.05 after FDR adjustment), 10% methylation difference and computed with RADmeth (Dolzhenko and Smith)^14^. Single replicate DMPs were called with Fisher's Exact Test after Benjamini-Hochberg FDR adjustment (*P*<0.05) with minimum 10% methylation difference and computed with custom software. Differentially methylated regions (DMRs) were defined as iCGs with 10% minimum methylation difference and at least 3 DMPs per region and computed using BSmooth (Hansen *et al*.)^10^. Blocks of comethylation (COMETs) were defined and computed using COMETgazer and differentially methylated COMETs (DMCs) were defined and computed using COMETvintage. The workflow for COMET analysis is shown in Supplementary Fig. 1 and discussed in the section *Workflow and feature definitions*. Here we describe the definition of COMETs, and their relative count distributions as follows:

### Definition of blocks of co-methylation (COMETs): the COMETgazer algorithm

We define the stochastic *oscillator* of *methylation* (**OM**) (Fig. 1a) as the one-series percentage change of CpG methylation (estimated on beta values, based on smoothed count data) in a single sample calculated as follows:


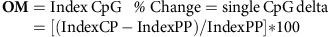


where IndexCP defines current CpG beta value and IndexPP is previous (upstream) CpG beta value. The harmonics define the segmentation of the COMETs in a sequential manner across chromosomes treating the data as if it were a time series. This definition is inspired by Ulrich *et al*. (2013) (ref. 32) and Ryan *et al*. (2014) (ref. 33), applications for calculating K series % changes for stock variation in financial modelling. The relationship between methylation levels and delta OM values is shown for a representative region in Fig. 1b.

COMETs are thus calculated using the following COMETgazer algorithm:

1. Define the CpG data points: by definition only canonical iCG are taken into consideration
2. Smooth CpG methylation (beta) scores
3. Calculate OM (Single CpG delta) globally
4. Define COMETs as regions of contiguous iCG where OM_n_ and OM_n+1_ oscillate around 0, with the arbitrary parameter (threshold) of dynamic oscillation termed **OMg** (oscillator of methylation grade) set to be +/− 0.1 OM over smoothed beta scores (at least +/− 10% methylation difference), roughly representing 8% of the delta (OM) distribution. This step is illustrated in Fig. 1c,d.

### Definition of OORTcloud distributions and the COMETvintage algorithm

Observed Oscillatory Rhythm Transition of COMET Longitudinally Obtained Undulation Domains (OORTcloud) was calculated by binning COMETs over 100,00 bp windows at each methylation level (high: hCOMET, medium: mCOMET, low: lCOMET) as shown in Supplementary Fig. 3. In this manner, we created three distributions of COMET domains.

For the DMC analysis, OORTcloud distributions were built in a count matrix in R. Differential methylome structure as defined by sample COMET counts was assessed with a negative binomial model using replicate values for the two samples (M1–M2, i.e., monocytes and the individual hESC replicates M7–10) using the *Bioconductor* package *edgeR* (Robinson *et al*.)^34^. Statistical significance is taken to be at *P*<0.05. An example of DMP, DMR and COMET comparison is shown in Fig. 3b and Supplementary Fig. 4.

### Workflow and feature definitions

The workflow for COMET analysis (https://github.com/rifathamoudi/COMETgazer) is shown in Supplementary Fig. 1, and involves the following 3 steps:

**Step 1** (tool: **COMETgazer**)

Key feature: COMET, i.e., region of co-methylation

Process: For each methylome, **individual CpG methylation level (beta) distributions** were used to compute **OM scores** and segment samples into **COMETs.**

Result: profiling the structure of the methylome

**Step 2** (tool: **COMETvintage**)

Key feature: OORTcloud distributions, i.e., distributions of COMET counts

Process: For each methylome, **COMETs** were binned into **count distributions** reflecting the **COMET domains (OORTcloud).**

Result: profiling the distribution of COMET counts across the methylome

**Step 3** (tool: **COMETvintage**)

Key feature: Differentially methylated COMETs (DMC)

Process: For differential methylation analysis, **COMET domains (OORTcloud)** were assembled into a **count matrix** to call regions of **differential methylated COMET (DMC)** counts.

Result: assessing DMCs between methylomes.

### Relationship between COMETs and linkage disequilibrium

Data were normalized to a 0–1 scale in order to compare COMETs with linkage disequilibrium (LD). Haplotype blocks for the sample Coriell NA18507 (HapMap GM18507) representing an African (YRU) haplotype and HapMap data for an European haplotype (CEU) were defined using a r^2^ threshold of 0.9 and calculated by binning the data into 100 bp windows and estimating the coverage counts of these over 100,000 bp windows for three distributions (depending on size) using two quantile thresholds (33%, 66%). In this manner, we obtained values for large (laHAB), medium (meHAB) and small haplotype blocks (smHAB) for each of the windows. The same procedure was applied to COMETs, obtaining counts for large (laCOMET), medium (meCOMET) and small (smCOMET) COMET by size. For each window, the normalized COMET score (NC) and normalized haplotype block score (NH) was obtained as follows:


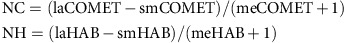


The scores for haplotype blocks and COMETs obtained were rescaled to a 0–1 distribution for a direct comparison. The genome was segmented according to haplotype block size into 10 quantile regions. For each segment, Pearson correlation analysis was carried out in order to estimate the significance of the relationship between median haplotype block size and median COMET size (Fig. 3c) representing an African (YRU) haplotype and Supplementary Fig. 6 representing an European (CEU) haplotype.

Multiple combinations of OMg and r^2^ were used. An example region for OMg=0.1 and r^2^>0.9 is illustrated in Supplementary Fig. 5. The correspondence between haplotype block size and COMETs is likely to be driven by haplotype blocks overlapping multiple regions of gene body methylation: large haplotypes (> quantile 66%) overlap with gene bodies at high methylation levels (quantile>66% of our WGBS data) and high gene expression (> quantile 66% of merged replicate data for exon array of GM18507, i.e., wgEncodeDukeAffyExonGm18507 downloaded from University of Santa Cruz Genome Browser) by 75% (*P*<0.0001, hypergeometric test).

### Code availability

COMETgazer is available for download at *https://github.com/rifathamoudi/COMETgazer*.

## Additional information

**How to cite this article:** Libertini, E. *et al*. Information recovery from low coverage whole-genome bisulfite sequencing. *Nat. Commun.* 7:11306 doi: 10.1038/ncomms11306 (2016).

## Supplementary Material

Supplementary Information Supplementary Figures 1-6, Supplementary Tables 1-4, Supplementary Notes 1-2 and Supplementary References
