# Supplementary material for: Information recovery from low coverage whole-genome bisulfite sequencing
Source: Nat Commun. 2016 Jun 27;7:11306. doi: 10.1038/ncomms11306 (PMC4931220; doi:10.1038/ncomms11306)
Supplement: Supplementary Information — Supplementary Figures 1-6, Supplementary Tables 1-4, Supplementary Notes 1-2 and Supplementary References [file ncomms11306-s1.pdf]

Supplementary Figures

Supplementary Figure 1.

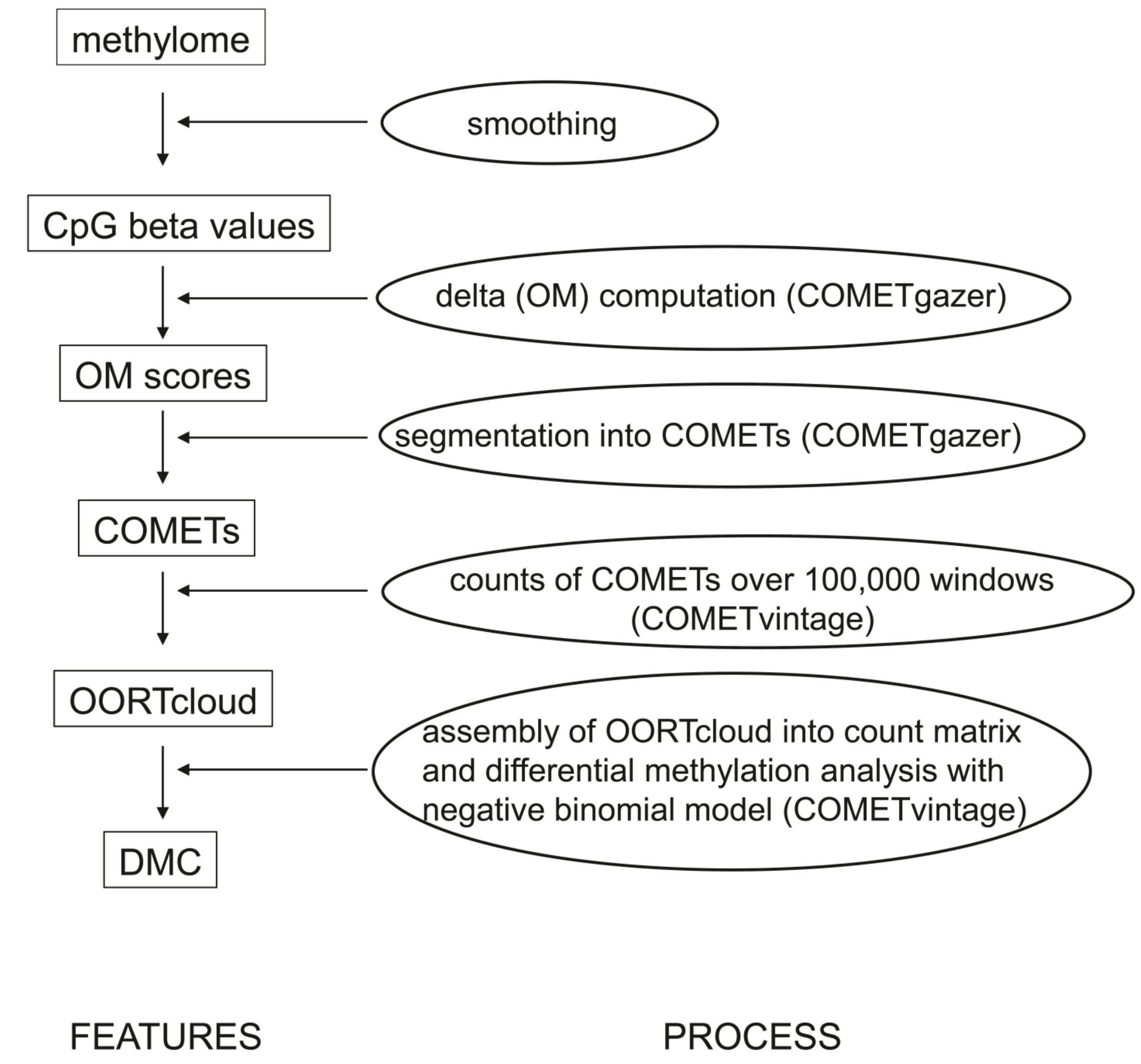

Supplementary Figure 1. COMET analysis workflow. With COMETgazer, methylome segmentation is determined by profiling COMETs. For each methylome, beta distributions were used to calculate OM scores, and profile COMETs. With COMET vintage, COMETs were counted at each methylation level (high, medium and low) in 100,000 bp windows, resulting in OORTcloud distributions. For differential methylation analysis, COMET domains (OORTcloud) were assembled into a count matrix for DMC calling.

Supplementary Figure 2.

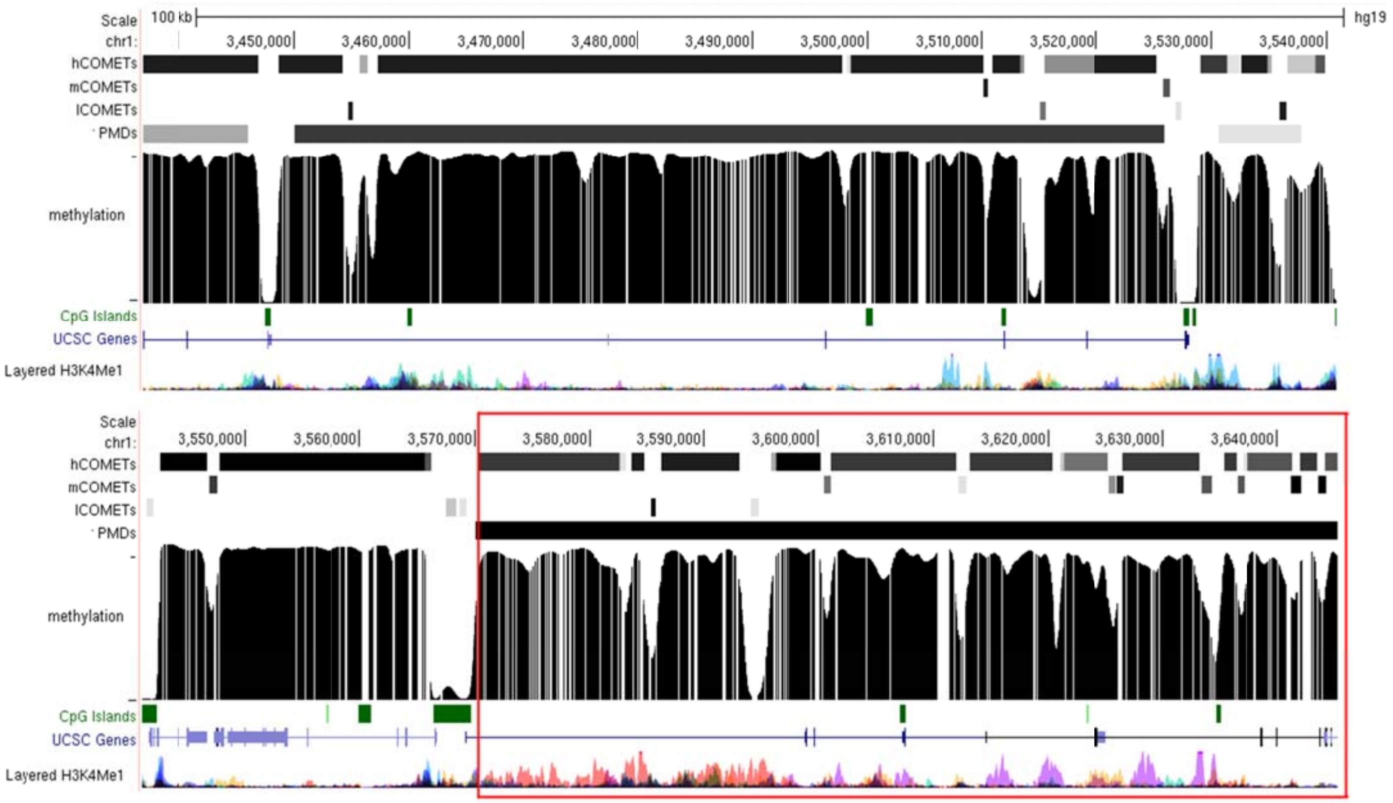

Supplementary Figure 2. COMET and PMD segmentation with corresponding methylation values. Data from M1 were used to show the overlap of PMDs with layered H3K4me1 signal (ENCODE) and the corresponding COMET tracks. The red box highlights a PMD region with its COMET structure breakup and corresponding layered H3K4Me1 signal. COMET shading in grey corresponds to average methylation value. PMD shading in grey corresponds to PMD size.

Supplementary Figure 3.

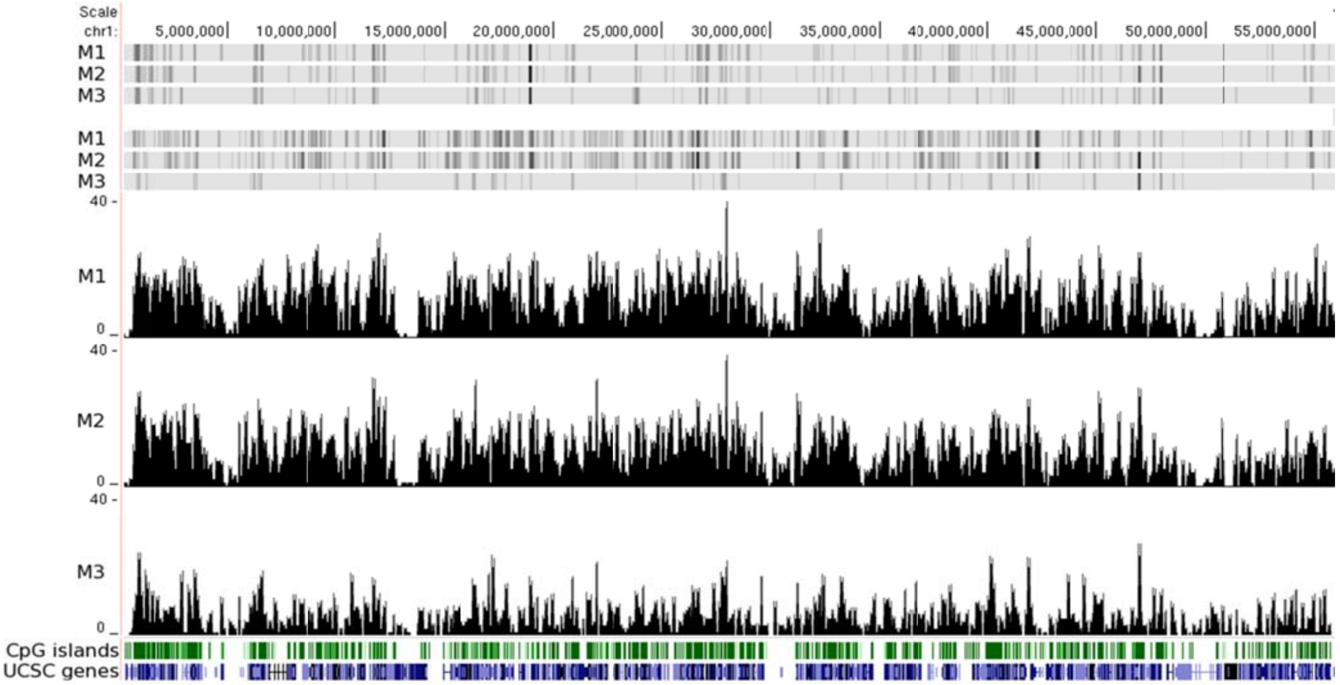

Supplementary Figure 3. OORTcloud distributions for the deep methylomes under investigation (M1-3). Counts for lCOMET (regions of low methylation level, top), mCOMET (partially methylated domains, middle) and hCOMET (regions of high methylation level, bottom) for each of the methylomes across the p arm of chromosome 1. OORTcloud distributions are a feature for complexity reduction of methylome structure, highlighting patterns of similarity at high dimensional scale. Tracks are shaded in grey to a maximum of 40 COMETs per window.

Supplementary Figure 4.

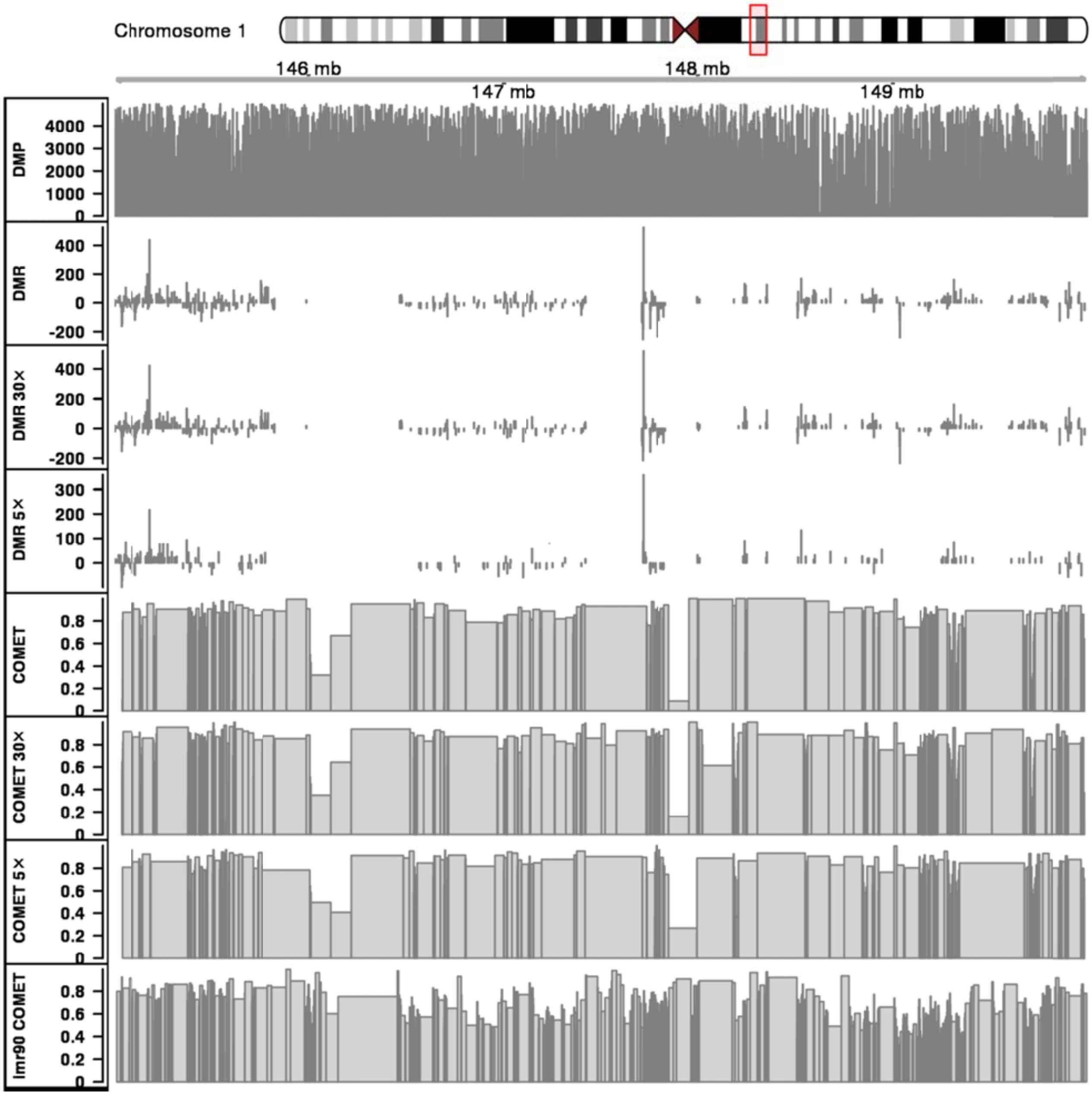

Supplementary Figure 4. Example of DMP, DMR and COMET reproducibility. Histogram plots along the chromosome 1 illustrating differences in DMR detection and COMET structure between M1-2 and M4. DMP values correspond to adjusted p-values multiplied by 100,000. DMR values correspond to the BSmooth *areaStat* parameter. COMETs are shown at average methylation level. It is interesting to note the reproducibility of COMET structure at each of the coverage (maximum, 30X, 5X). This figure also highlights the contrast between point-wise differences (DMP) and DMR, which are grouped and represent only a subset of overall differential methylation.

Supplementary Figure 5.

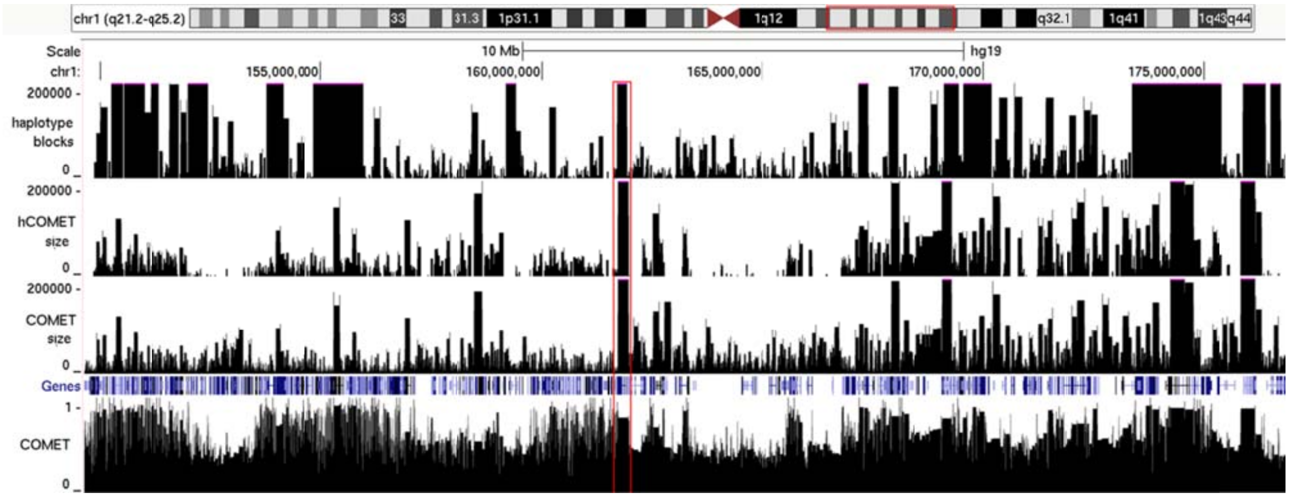

Supplementary Figure 5. Relationship between haplotype block size (defined by linkage disequilibrium with a threshold of $r^2 > 0.9$) and COMET size (defined by OMg = 0.1) for M5. Haplotype blocks, COMET and hCOMET size values correspond to their size in base pairs. A track for M5 COMETs is shown at their average methylation value. A typical example of size and coordinate correspondence is highlighted (red box).

Supplementary Figure 6.

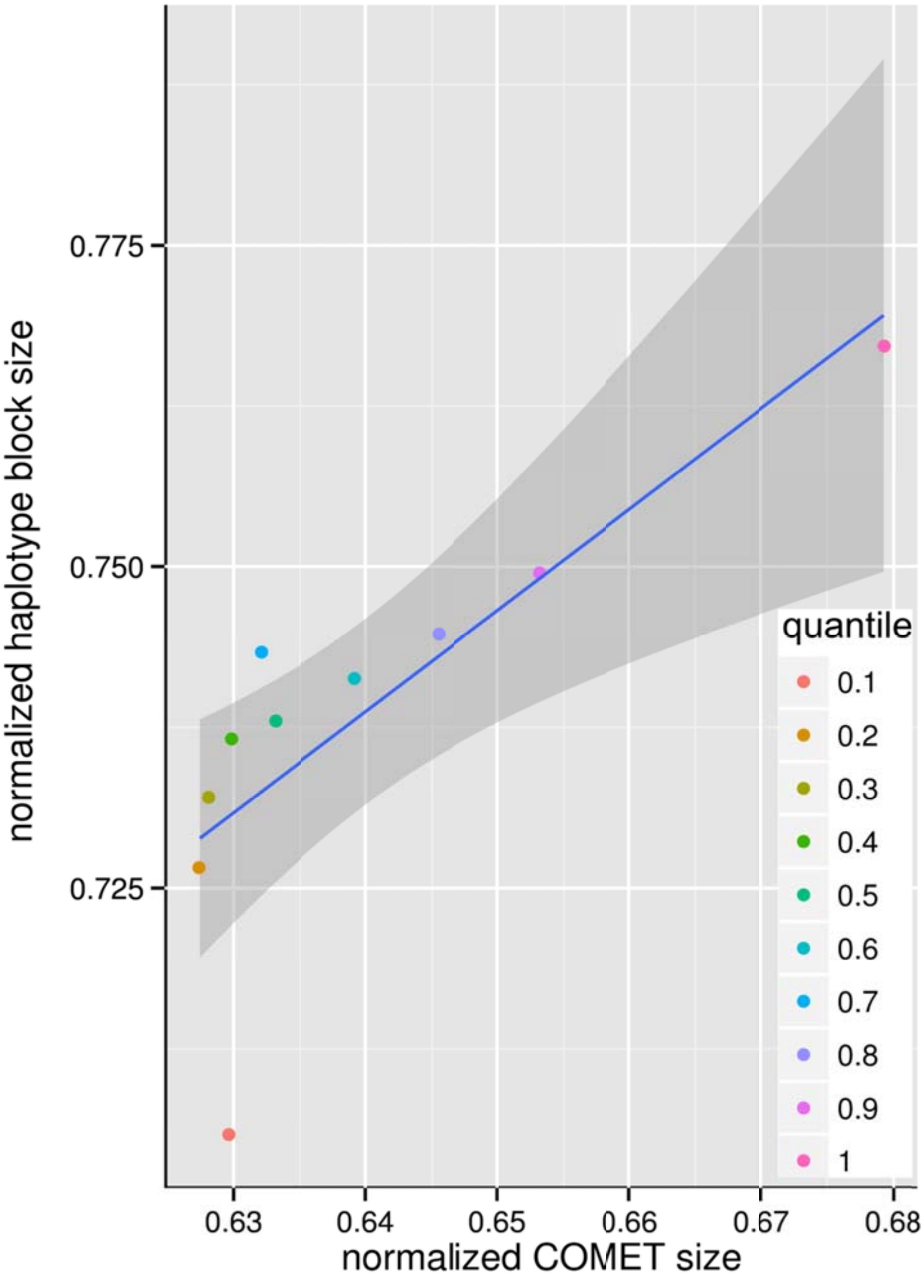

Supplementary Figure 6. Correlation between CEU haplotype blocks and YRU COMETs. Median haplotype block size defined by $r^2 > 0.9$ versus median COMET size for M5 (representative of an African population). Data was tiled over fixed windows of 100,000 bp and scaled over 0-1 (**Supplementary Information, Methods**).

Supplementary Tables

Supplementary Table 1.

methylome	source	cell type	accession number	million read-pairs	yield (Gb)	trimmed yield (Gb)	reference	% unique read pairs	bisulfite conversion efficiency	median coverage
**M1**	CNAG	monocytes	EGAD00001001261	1855.7	374.5	364	hg19	81.367	99.7	83
**M2**	CNAG	monocytes	EGAD00001001261	2043.8	412.5	401.1	hg19	81.7	99.7	91
**M3**	Broad Institute	embryonic stem cells (hESC)	GSM916051 GSM1112848 GSM1112840 GSM1112841	3391.1	339.1	294.1	hg19	88.1	99.5	91
**M4**	Lister et al. 2009	lung fibroblasts (imr90)	GSE17917	Lister et al. 2009	Lister et al. 2009	Lister et al. 2009	liftOver from hg18 to hg19	Lister et al. 2009	Lister et al. 2009	29
**M5**	Illumina	Coriell (Yoruba) NA18507 (HapMap GM18507)	GSE66285	5447.4	828	809.5	hg19	68.1	99.7	37
**M6**	CNAG	neutrophils	EGAN00001092671	962.0	192.4	187.0	hg19	81.8	99.6	40
**M7**	Broad Institute	embryonic stem cells (hESC)	GSM112840	531.6	53.2	48.1	hg19	90.5	99.4	22
**M8**	Broad Institute	embryonic stem cells (hESC)	GSM112841	762.7	76.3	66.8	hg19	87.5	99.4	28
**M9**	Broad Institute	embryonic stem cells (hESC)	GSM916051	691.1	69.1	59.1	hg19	85.4	99.6	17
**M10**	Broad Institute	embryonic stem cells (hESC)	GSM1112848	1405.5	140.5	120	hg19	85.5	99.6	45
**M11**	Broad Institute	embryonic stem cells derived from CD56+ mesoderm	GSM1112839	Ziller et al. 2013	Ziller et al. 2013	Ziller et al. 2013	hg19	Ziller et al. 2013	Ziller et al. 2013	33
**M12**	Broad Institute	embryonic stem cells derived from CD56+ mesoderm	GSM112842	Ziller et al. 2013	Ziller et al. 2013	Ziller et al. 2013	hg19	Ziller et al. 2013	Ziller et al. 2013	19
**M13**	BGI	peripheral blood mononuclear cells	GSE17972	Li et al. 2010	Li et al. 2010	Li et al. 2010	liftOver from hg18 to hg19	Li et al. 2010	Li et al. 2010	25
**M14-M15**	UCL	monocytes	replicated data for simulated RRBS coordinates was generated from M1 and M2							

Supplementary Table 1. Summary of methylomes included in the analysis. This table includes a summary of data sets and quality measures for all methylomes used in this study.

Supplementary Table 2.

methylome	cell type	COMETs	median COMET length
M1	monocytes	225188	904 bp
M2	monocytes	216235	916 bp
M3	embryonic stem cells	112799	942 bp
M4	lung fibroblasts	416803	1647 bp
M5	lymphoblastoid	957612	1034 bp
M6	neutrophils	214791	886 bp
M7	embryonic stem cells	114243	1120 bp
M8	embryonic stem cells	118069	1034 bp
M9	embryonic stem cells	101471	1032 bp
M10	embryonic stem cells	105779	922 bp
M11	hESC derived CD56+ mesoderm	162819	1398 bp
M12	hESC derived CD56+ mesoderm	164225	1532 bp
M13	peripheral blood mononuclear cells	259007	1632 bp

Supplementary Table 2. Summary of COMET counts for the analyzed methylomes. The number of fragmentations per methylome is reported as number of COMETs together with the median length of the COMETs for each methylome. This table illustrates the overall distinct methylome structure between the cell types under investigation, as well as the reproducibility of COMET structure across biological replicates (M1-M2). Note that the M5 methylome (NA18507) is highly fragmented. Median COMET lengths are concordant with what was previously reported by Eckhardt et al. (2006) with respect to spatial DNA comethylation correlations.

Supplementary Table 3.

	hCOMETs	mCOMETs	lCOMETs	CGI	shores	PMD	LMR	UMR
**hCOMETs**	1	-0.2	-0.2	-0.1	-0.1	-0.2	-0.1	-0.2
**mCOMETs**	-0.2	1	0.1	0.1	0.1	-0.1	0.4	0.1
**lCOMETs**	-0.2	0.1	1	0.4	0.3	-0.1	0.1	0.7
**CGI**	-0.1	0.1	0.4	1	NA	-0.2	0	0.6
**shores**	-0.1	0.1	0.3	NA	1	-0.2	0.1	0.6
**PMD**	-0.2	-0.1	-0.1	-0.2	-0.2	1	-0.2	-0.2
**LMR**	-0.1	0.4	0.1	0	0.1	-0.2	1	0.1
**UMR**	-0.2	0.1	0.7	0.6	0.6	-0.2	0.1	1

Supplementary Table 3. Correlation matrix illustrating the relationship between features defined by MethylSeekR, COMETs and genomic features such as CGI and shores.

Supplementary Table 4.

Sample	OMg	rsq	correlation	significance
YRU	0.1	0.9	0.86	0.00112
YRU	0.1	0.95	0.50	0.1412
YRU	0.2	0.9	0.65	0.0435
YRU	0.2	0.95	0.82	0.003343
CEU	0.1	0.9	0.80	0.004871

Supplementary Table 4. Summary of sample and parameter combinations comparing COMET size and haplotype block size. For each combination, the parameters defining COMETs and haplotype blocks were changed. These include oscillations of methylation grade (OMg), defining COMETs (0.1 or 0.2) and r^2, defining haplotype blocks (0.9 or 0.95). The resulting correlation between the size of COMETs and haplotype blocks is reported for each combination.

Supplementary Notes

Supplementary Notes 1: *Samples*

Methylomes M1 and M2 were obtained from purified monocytes. Monocytes were purified (>95% pure) from blood donors of the Cambridge BioResource after informed consent was obtained (NRES Committee East of England-Hertfordshire, 12/EE/0040). Whole blood was separated by gradient centrifugation and monocytes (CD14+ CD16-) were further isolated from the mononucleated layer by negative CD16 selection followed by positive CD14 selection. All samples underwent flow cytometry, morphological and expression array analysis. The full protocol is available at:
http://www.blueprint-epigenome.eu/UserFiles/file/Protocols/UCAM_BluePrint_Monocyte.pdf.
M3 was obtained from four human embryonic stem cell replicates (M7-10) described in Ziller et al. (2013)[1]. M11-M12 were obtained from human embyronic stem cells derived from CD56+ mesoderm cells described in Ziller et al. (2013)[1].
M4 was obtained from the lung fibroblast (imr90) cell line described in Lister et al. (2009)[2]. Data were not realigned; a lift over tool was used to convert the data to hg19 coordinates. Likewise, M13 was obtained from the peripheral blood mononuclear cell methylome described in Li et al. (2010)[3] and hg18 data were converted to hg19. M5 was derived from Coriell's lymphoblastoid cell line (NA18507).

Supplementary Notes 2: *Library preparation and sequencing*

Library preparation and sequencing of M1 and M2 was conducted at the Centre Nacional d'Anàlisi Genòmica as described in (Kulis et al, 2012)[4]. Briefly, the libraries were generated from 2 µg of genomic DNA. This was spiked with unmethylated λ DNA (Promega) at a concentration of 5 ng of λ DNA per 1 µg of genomic DNA. The short-insert paired-end library was prepared using the TruSeq™DNA Sample Preparation Kit v2 (Illumina Inc.) and the KAPA Library Preparation kit (Kapa Biosystems). In brief: The DNA was sheared with a Covaris E220 (Covaris) to 50–500 bp and size-selected to 150-300 bp fragments using AMPure XP beads (Agencourt Bioscience Corp.). Using the KAPA Library Preparation kit the DNA fragments were end-repaired, adenylated and ligated to Illumina specific indexed paired-end adaptors. After adaptor ligation, the DNA was treated with sodium bisulfite using the EpiTect Bisulfite kit (Qiagen) following the manufacturer's instructions with two rounds of conversions. After bisulfite conversion the adaptor-ligated DNA was amplified with 7 cycles of PCR using the PfuTurboCx Hotstart DNA polymerase (Stratagene). The library was quality controlled using BioAnalyzer 7500 assay (Agilent). The library was sequenced on HiSeq2000 (Illumina, Inc.) following the manufacturer’s protocol, in paired end mode with a read length of 2x101bp in 11 sequencing lanes. Images analysis, base calling and quality scoring of the run were

processed using the manufacturer's software Real Time Analysis (RTA 1.13.48). For M3, M7-10, M11-12 library preparation and sequencing was as described in Ziller et al. (2013)[1] and for M4 in Lister et al. (2009)[2].

For M5, library preparation and sequencing was conducted at Illumina Inc., San Diego. Briefly, the library was derived from 100 ng of Coriell's lymphoblastoid gDNA (NA18507), which was treated with EZ DNA Methylation-Lightning bisulfite conversion kit (Zymo Research, USA) according to the manufacturers' recommendations. The resulting DNA was used to prepare whole-genome bisulfite library as described in the Illumina's EpiGnomeTM Methyl-Seq Kit manual. Briefly, bisulfite-treated single-stranded DNA undergoes subsequent DNA synthesis, terminal tagging, amplification, library purification, quantification and cluster generation. The EpiGnome library concentration was measured to be 17ng/ul using the Qubit HS kit (Life Technologies, USA) with a median library size of 361bp on a Bioanalyzer High Sensitivity DNA chip (Agilent Technologies Inc., USA). A single library was then diluted to 10 pM and sequenced with 75 base paired-end reads, on 30 flowcell lanes, using an Illumina HiSeq 2500 instrument in high output run mode. In order to assess the quality of the run 1% PHIX (Catalog # FC-110-3001) was spiked into the library prior sequencing. Error rates were less than 1% and the quality scores were on average 95% over Q30.

Supplementary References

1. Ziller, M.J. *et al*. Charting a dynamic DNA methylation landscape of the human genome. *Nature*. **500**, 477-81 (2013).

2. Lister, R. *et al.* Human DNA methylomes at base resolution show widespread epigenomic differences. *Nature*. **462**, 315-22 (2009).

3. Li, Y., *et al*. The DNA methylome of human peripheral blood mononuclear cells. PLoS Biol. **8**, e1000533 (2010).

4. Kulis M., Heath S., Bibikova M., Queirós A.C., Navarro A. *et al.* Epigenomic analysis detects widespread gene-body DNA hypomethylation in chronic lymphocytic leukemia. *Nat. Genet.* **44**, 1236-42 (2012).
